# Anesthetic Management of a Pregnant Woman With an Unruptured Cerebral Aneurysm Undergoing Simultaneous Cesarean Section and Craniotomy for Aneurysm Clipping: A Case Report

**DOI:** 10.1155/cria/2681007

**Published:** 2025-12-23

**Authors:** Kakeru Okubo, Yui Ikuta, Erika Imasato, Sayaka Kawamura, Hideya Kato

**Affiliations:** ^1^ Department of Anesthesiology, Saiseikai Shigaken Hospital, Ritto, Shiga, Japan

**Keywords:** aneurysm clipping, cesarean section, pregnancy, transmural aneurysmal pressure, unruptured cerebral aneurysm

## Abstract

Unruptured cerebral aneurysms during pregnancy carry a high risk of rupture in late gestation. Simultaneous cesarean section and aneurysm clipping are rare and demand careful anesthetic planning. A 34‐year‐old woman at 36‐week gestation presented with progressive oculomotor palsy caused by an unruptured 8‐mm cerebral aneurysm. A sequential cesarean delivery, followed by aneurysm clipping, was planned. Spinal anesthesia was used for cesarean section to minimize fetal sedation and then converted to general anesthesia for neurosurgery. Transmural aneurysmal pressure (TAP) was maintained by avoiding mean arterial pressure surges and excessive intracranial pressure reduction. The procedure was uneventful, and both the mother and neonate recovered without any complications. This case highlights the importance of multidisciplinary planning and TAP‐guided hemodynamic control in achieving favorable maternal and fetal outcomes in high‐risk pregnancies.

## 1. Introduction

Cerebral aneurysm rupture during pregnancy is associated with high maternal and fetal mortality rates, with increased risk in late gestation due to hormonal and circulatory changes [1, 2]. Anesthetic management must prioritize maternal safety, while minimizing fetal risk. We report a rare case of combined cesarean section and aneurysm clipping, emphasizing transmural aneurysmal pressure (TAP)–guided hemodynamic management and multidisciplinary coordination.

## 2. Case Presentation

**Figure 1:**
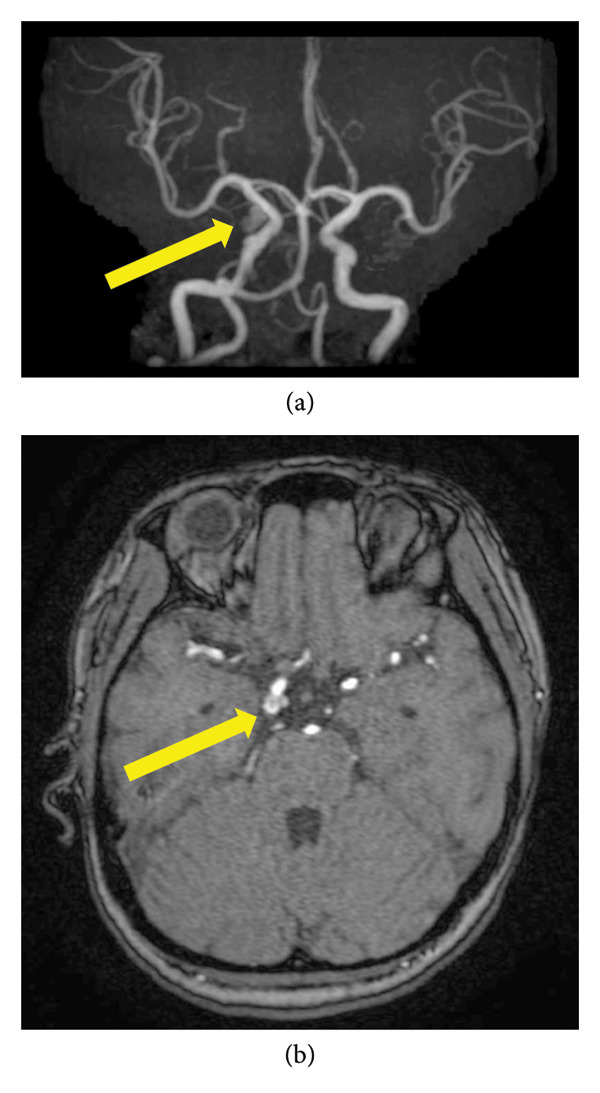
Preoperative brain magnetic resonance imaging findings. An 8‐mm cerebral aneurysm can be seen at the right internal carotid artery–posterior communicating artery bifurcation. (a, b) Axial section (a) and magnetic resonance angiography (b) showing the aneurysm (yellow arrows).

A 34‐year‐old Gravida 3, Para 1, medically unremarkable, began experiencing right‐sided pulsatile headaches at 35 weeks of gestation and developed visual disturbances at 36 weeks and 3 days. That evening, she developed diplopia and right‐sided ptosis, prompting referral to our hospital at 36 weeks and 4 days. On admission, her vital signs were stable, with blood pressure 126/77 mmHg, heart rate 64 beats/min, and oxygen saturation 96% in room air. Brain magnetic resonance imaging (MRI) revealed an 8‐mm unruptured cerebral aneurysm at the right internal carotid artery–posterior communicating artery bifurcation (Figure 1). Multidisciplinary discussion among obstetricians, neurosurgeons, and anesthesiologists concluded that (1) progressive symptoms and oculomotor palsy suggested enlargement and impending rupture, (2) risk of rupture increased in late pregnancy with blood pressure fluctuations during labor, and (3) the fetus was viable. Based on these factors, the emergency cesarean section was performed owing to the high risk of rupture and the presence of progressive oculomotor symptoms, which indicated impending aneurysm rupture. Thus, a sequential procedure combining cesarean delivery and aneurysm clipping was deemed optimal.

### 2.1. Anesthetic Management

**Figure 2:**
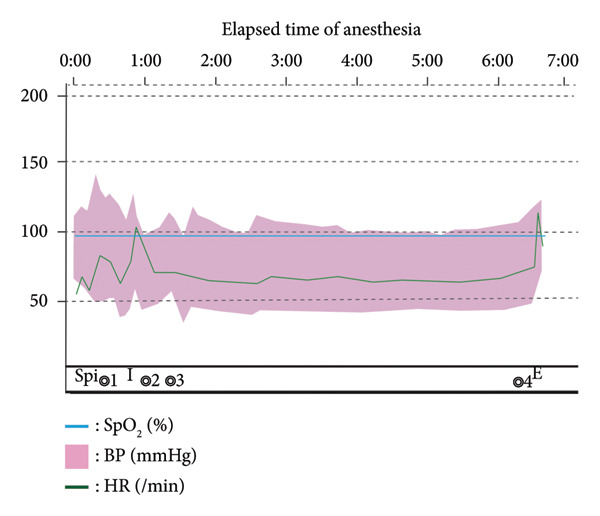
Anesthesia records. 0:06 insertion of the arterial line (right radial artery). 0:14 spinal anesthesia. 0:31 ◎1: start of obstetric surgery. 0:36 delivery of the baby. 0:54 I: tracheal intubation and initiation of general anesthesia. 1:01 ◎2: end of obstetric surgery. 1:37 ◎3: start of neurosurgery. 6:33 ◎4: end of neurosurgery. 6:43 E: extubation. 6:50 exit from the operating room. BP, blood pressure; HR, heart rate; SpO_2_, peripheral capillary oxygen saturation.

Approximately 3 h after MRI, the patient was brought to the operating room. Under invasive arterial pressure monitoring, spinal anesthesia was administered at L3‐4 using 2.4 mL of 0.5% hyperbaric bupivacaine and 15 μg fentanyl. A cold test confirmed a sensory block below T4, and cesarean section was initiated. After delivery, general anesthesia was induced using propofol, remifentanil, and rocuronium, followed by tracheal intubation. The neonate had Apgar scores of 9 at 1 and 5 min. Oxytocin was administered slowly over 3 h after delivery to promote uterine contractions, while minimizing hemodynamic fluctuations. Anesthesia was maintained with desflurane and remifentanil. During neurosurgery, the blood pressure was controlled to avoid abrupt changes. Although intracranial pressure (ICP) was not directly monitored, TAP was managed by preventing mean arterial pressure (MAP) surges and avoiding hyperventilation or excessive cerebrospinal fluid (CSF) drainage. The aneurysm was successfully clipped without rupture. The patient was extubated and returned to the ward. The timeline is shown in Figure 2. Postoperative analgesia was provided via intravenous patient‐controlled analgesia and nonsteroidal anti‐inflammatory drugs. Imaging revealed no residual aneurysms or new lesions. Preexisting oculomotor palsy improved, but persisted at discharge, and mecobalamin therapy was continued. No anesthetic complications occurred. Both the mother and child were discharged on postpartum Day 8. Written informed consent was obtained from the patient.

## 3. Discussion

The prevalence of unruptured cerebral aneurysms in the general population is approximately 3% [3]. In Japan, the incidence of aneurysmal subarachnoid hemorrhage is relatively high, at 28 per 100,000 people [4]. During pregnancy, increased blood volume, cardiac output, and hormonal changes increase the risk of rupture [1, 2]. Among ruptured aneurysms during pregnancy, most occur in the third trimester [1]. Maternal mortality ranges from 13% to 35% and fetal mortality, from 7% to 25%, emphasizing the need for prevention [2]. However, no standardized guidelines exist for managing unruptured aneurysms in pregnancy, and clinical experience is limited [2, 5]. Even small aneurysms carry a high rupture risk and warrant proactive intervention [1]. Surgical timing depends on fetal maturity; when viability is limited, neurosurgery may precede delivery [6]. In sequential surgery, no clear guidelines exist on which procedure to perform first [2]. In this case, emergency neurosurgery was necessary due to an impending rupture. As the fetus was viable, cesarean delivery, followed by clipping, was performed.

For sequential procedures, general anesthesia alone or regional–general combinations may be used. TAP, the difference between MAP and ICP, is critical. Elevated TAP heightens rupture risk; thus, both MAP elevation and ICP reduction must be avoided [7, 8]. General anesthesia avoids conversion during surgery but raises concerns about fetal sedation and respiratory depression (“sleeping baby” phenomenon) from placental drug transfer. Pregnancy also increases the risk of difficult intubation and aspiration, and intubation‐related blood pressure surges threaten aneurysm stability. Fetal monitoring is necessary if neurosurgery precedes delivery [2]. Spinal anesthesia avoids fetal sedation but may lower ICP due to CSF leakage, theoretically increasing TAP and rupture risk [7, 8]. Although safely used in many cases, its effect on rupture risk remains unclear [7, 8]. Epidural anesthesia avoids fetal sedation, and increased epidural pressure may increase ICP and lower TAP, but inadvertent dural puncture can negate this [5]. Given the tradeoffs, no standard strategy exists; each case requires tailoring. Excessive TAP reduction can dangerously lower cerebral perfusion pressure; therefore, extreme MAP reduction or ICP elevation must be avoided [9]. The TAP theory has limitations due to the difficulty in measuring ICP in pregnancy.

In this case, spinal anesthesia was chosen to avoid fetal sedation and allow maternal–fetal bonding, with preparations for immediate general anesthesia if rupture occurred. Neurosurgeons were on standby, and invasive arterial monitoring was performed before spinal anesthesia to enable precise control. Intubation was performed using rapid sequence induction, considering airway edema and reduced functional residual capacity. Oxytocin, a uterotonic agent, theoretically reduced TAP through vasodilation [7]. To avoid abrupt circulatory changes, it was administered slowly under uterine tone monitoring. Aneurysm treatment should prioritize maternal outcomes, as optimizing the maternal condition also improves fetal outcomes [10, 11]. In cases where the treatment occurs at a gestational age compatible with survival, multidisciplinary collaboration is vital [2]. Outcomes do not differ significantly between clipping and endovascular treatment for ruptured aneurysms in pregnancy [10]. Fetal radiation exposure is generally safe with precautions [10]. However, antithrombotic use may increase bleeding risk in emergency cesarean delivery, and logistic challenges exist in angiography suites [1, 7]. Vaginal deliveries have been reported, but Valsalva maneuvers may increase ICP, MAP, and TAP [1, 7]. Vaginal delivery with epidural analgesia may minimize TAP fluctuations. Simultaneous cesarean delivery and aneurysm surgery under coordinated anesthetic management is rare in Japan. This case applies recent knowledge, including the TAP theory and benefits of regional anesthesia, and may inform future risk assessment models.

In conclusion, this report described the successful anesthetic management of a patient undergoing combined cesarean section and craniotomy at 36 weeks and 4 days with impending aneurysm rupture. Spinal anesthesia was used for cesarean delivery, followed by general anesthesia for clipping. Intraoperative hemodynamics remained stable, and no rupture occurred. Early detection, intervention, and interdisciplinary coordination are keys to favorable outcomes. This rare case offers insights into the perinatal management of unruptured aneurysms and demonstrates that well‐planned sequential surgery can result in favorable outcomes. Notably, it explicitly integrates TAP‐guided management into a sequential procedure [7, 8]. This multidisciplinary are warranted to determine optimal anesthetic approaches and TAP strategies in this population.

## Conflicts of Interest

The authors declare no conflicts of interest.

## Funding

No financial support was provided for this study.

## Data Availability

The datasets used in this study are available from the corresponding author upon request.
